# Volatile organic compound data of ready-to-cook tuna fish-burgers: Time evolution in function of different and/or combined mild preservation technologies and relevant statistical analysis

**DOI:** 10.1016/j.dib.2019.104371

**Published:** 2019-08-07

**Authors:** Annalisa Mentana, Amalia Conte, Matteo Alessandro Del Nobile, Maurizio Quinto, Diego Centonze

**Affiliations:** a SAFE — Department of Science of Agriculture, Food and Environment, University of Foggia, via Napoli 25, I-71122 Foggia, Italy; b Key Laboratory of Natural Resources of the Changbai Mountain and Functional Molecules, Yanbian University, Park Road 977, Yanji 133002, Jilin Province, PR China

**Keywords:** Volatolomics, HS-SPME-GC-MS, Ready-to-cook fish burger, Modified atmosphere packaging, Preservation mild technologies

## Abstract

Volatile organic compound (VOC) composition from ready-to-cook tuna fish-burgers, prepared with and without a protective microbial strain (Lactobacillus paracasei) and/or stored with modified atmosphere packaging (MAP, 5% O2 and 95% CO2), were extracted by headspace solid-phase microextraction and analyzed by gas chromatography-mass spectrometry (HS-SPME-GC-MS) during the burger shelf-life. The collected data showed volatile composition profiles in function of the mild preservation technologies employed and the storage time. Furthermore, statistical data treatment (principal component analysis and Pearson's coefficients) highlighted differences among samples and positive/negative correlations during the storage time. This paper is related to an article already published in LWT (Investigating the effects of mild preservation technology on perishable foods by volatolomics: The case study of ready-to-cook tuna-burgers” https://doi.org/10.1016/j.lwt.2019.108425).

**Table:** Specifications table

Subject	Food Science, Analytical Chemistry
Specific subject area	Analytical Chemistry applied to Food Technology
Type of data	Table
How data were acquired	104 compounds were extracted by headspace solid-phase microextraction (HS-SPME) from ready-to-cook tuna fish-burgers previously treated with different mild preservation technologies (namely, Lactobacillus paracasei addition, packaging with 5% O_2_ and 95% CO_2_, and a combination of them) and successively identified by gas chromatography-mass spectrometry (GC-MS). Statistical multivariate approaches were used to highlight differences among treated and untreated samples.
Data format	Raw Analyzed
Parameters for data collection	Ready-to-cook tuna fish-burger preparation, sampling, extraction and analysis parameters are briefly described in this paper and fully provided in the related research article.
Description of data collection	Data were collected by a Agilent 5975 mass spectrometer detector hyphenated with an Agilent 6890 N gas chromatograph (Little Falls, DE, USA). The GC was coupled with a Gerstel MPS autosampler (Gerstel, Baltimore, MD, USA).
Data source location	Ready-to-cook tuna fish-burgers were prepared in our laboratory (Foggia) using frozen yellowfin tuna fillets (Thunnus albacares), provided by a local fishing company (Caggianelli, Manfredonia, Italy). City/Town/Region: Manfredonia Country: Italy
Data accessibility	Repository name: Mendeley Data identification number: https://doi.org/10.17632/9zghyckn85.3 Direct URL to data: https://data.mendeley.com/datasets/9zghyckn85/3
Related research article	Authors: Annalisa Mentana, Amalia Conte, Matteo Alessandro Del Nobile, Maurizio Quinto, Diego Centonze Title: Investigating the effects of mild preservation technology on perishable foods by volatolomics: the case study of ready-to-cook tuna fish-burgers Journal: LWT Food Science and Technology, LWT 115 (2019) 108425 https://doi.org/10.1016/j.lwt.2019.108425

**Table:** 

**Value of the data** • VOCs data give an insight on the effects of mild preservation technology and storage time on perishable foods • It has been demonstrated that volatolomics has the potential to improve the control quality of processed fresh food • A HS-SPME-GC-MS method to evaluate the volatile composition of tuna fish burgers has been described in details • HS-SPME-GC-MS can be used for fingerprinting analysis • Effect of modified atmosphere packaging (MAP, 5% O_2_ and 95% CO_2_) and or/protective microbial strain (*Lactobacillus paracasei*) on the volatile organic components on ready-to-cook fish burger

## 1 Data

All tables are reported in Ref. [1].

Table 1 shows the normalised peak area of VOCs isolated from tuna fish-burgers treated with different mild preservation technologies, in function of storage time, together with the analysis of variance (ANOVA). To investigate possible relationships between VOCs evolution and product quality, Pearson's coefficients related to compounds and chemical class peak areas are reported in table 2 and table 3, respectively. Finally, To highlight differences among volatile compositions of tuna-burgers as a function of storage time, scores of the first, second and third principal component analysis of each compound were provided by table 4, while table 5 displays scores and loadings of the first and second principal component analysis of the relevant chemical classes.

## 2 Experimental design, materials, and methods

### 2.1 Fish-burger preparation

A detailed description of fish-burger preparation is provided in the related research article. Briefly, frozen yellowfin tuna fillets (*Thunnus albacares*) were frozen at −18 °C. Before use, Tuna slices were thawed at 5 °C for 24 hours and then minced with an industrial food processor. Tuna fish burgers ingredients were as follows (g per kg of raw material): minced fish (765), extra-virgin olive oil (100), sodium chloride (5), parsley (5), rosemary (5), curry powder (5), potato starch (50) and potato flakes (65). For the inoculum preparation, bacterial cultures of protective microbial strain (*Lactobacillus paracasei*) grown in MRS-Broth (37 °C for 24 h) were centrifuged at 10000 g for 15 min at 4 °C. The pellets were re-suspended in sterile water at 4 °C and the resulting cell suspensions (9 Log cfu mL^−1^) were added to fishery dough prior to burger forming operation. Tuna-burgers without bacteria cultures were also prepared as the control. The tuna-burgers were packaged in commercially available bags (Nylon/Polyethylene) with thickness of 150 μm in air and under modified atmosphere packaging (MAP, 5% O_2_ and 95% CO_2_) and then kept under refrigeration.

### 2.2 Headspace sampling (HS-SPME) and gas chromatography-mass spectrometry (GC–MS) analysis

A Gerstel MPS autosampler (Gerstel, Baltimore, MD, USA) mounted on an Agilent 6890 N gas chromatograph (Little Falls, DE, USA) coupled with an Agilent 5975 mass spectrometer detector constituted the analytical system. The complete sampling procedure by HS-SPME and the GC-MS experimental conditions are described in the related research article. Briefly, 2.0 g of fish-burger were transferred into a 20 mL glass headspace sample vial together with 50 μL of internal standard solution (3-octanol, 20 ppm). The mixture was carefully shaken and then left 1 h in the dark at room temperature to equilibrate before the analysis.The SPME fiber coating used in this study was a 50/30 μm Divinylbenzene/Carboxen/Polydimethylsiloxane (DVB/CAR/PDMS) 23 gauge. Fish-burger samples were warmed to 40 °C for 15 min before exposing the SPME fiber to the headspace. HD-SPME sampling/extraction was carried out for 30 min at 40 °C, with continuous stirring at 250 rpm. SPME injections were made in splitless mode using a SPME injection sleeve (0.75 mm I.D.) at 250 °C for 350 sec. The column was an HP-INNOWax (60 m × 0.25 mm, 0.25 μm film thickness, J&W. Scientific, Folsom, USA). Helium carrier gas was used with a total flow of 1.0 mL min^−1^. The oven temperature was programmed to increase from 40 °C to 150 °C at 3 °C min^−1^ and maintained for 5 min, then increased to 200 °C at 15 °C min^−1^ and maintained at this temperature for 10 min before returning to the initial temperature. The MS detector operated in scan mode (mass range 35–350 amu) and the transfer line to the MS system was kept at 250 °C. Compound identifications were performed by comparing experimental mass spectrum to those contained in the National Institute of Standards and Technology database and by experimental linear retention indexes (LRI), based on a homologous series of n-alkanes.

### 2.3 Data analysis

Analysis of Variance (ANOVA) was carried out using XLSTAT (Addinsoft SARL, NY, USA) for Microsoft Excel (Microsoft, Redwood, WA) and Pearson's coefficients (r) were calculated in order to highlight correlations between compounds by using Microsoft Excel 2013.

## References

[1] Quinto M. (2019). Volatile Organic Compound Data of Ready-To-Cook Tuna Fish-Burgers: Time Evolution in Function of Different And/or Combined Mild Preservation Technologies and Relevant Statistical Analysis.

